# Peroxiredoxin 6 Is a Key Antioxidant Enzyme in Modulating the Link between Glycemic and Lipogenic Metabolism

**DOI:** 10.1155/2019/9685607

**Published:** 2019-12-19

**Authors:** Roberto Arriga, Francesca Pacifici, Barbara Capuani, Andrea Coppola, Augusto Orlandi, Maria Giovanna Scioli, Donatella Pastore, Aikaterini Andreadi, Paolo Sbraccia, Manfredi Tesauro, Nicola Di Daniele, Giuseppe Sconocchia, Giulia Donadel, Alfonso Bellia, David Della-Morte, Davide Lauro

**Affiliations:** ^1^Department of Systems Medicine, University of Rome “Tor Vergata”, Rome, Italy; ^2^Department of Anatomic Physiology, University of Rome “Tor Vergata”, Rome, Italy; ^3^Laboratory of Tumor Immunology and Immunotherapy, Institute of Translational Pharmacology, Department of Biomedicine, National Research Council (CNR), Rome, Italy; ^4^Department of Clinical Science and Translational Medicine, University of Rome “Tor Vergata”, Rome, Italy; ^5^San Raffaele Rome Open University, Rome, Italy; ^6^Evelyn F. McKnight Brain Institute, Department of Neurology, Miller School of Medicine, University of Miami, Miami, FL, USA; ^7^UOC of Endocrinology and Diabetes, University Hospital Fondazione Policlinico Tor Vergata, Rome, Italy

## Abstract

Insulin action and often glucose-stimulated insulin secretion are reduced in obesity. In addition, the excessive intake of lipids increases oxidative stress leading to overt type 2 diabetes mellitus (T2DM). Among the antioxidative defense systems, peroxiredoxin 6 (PRDX6) is able to reduce H_2_O_2_ and short chain and phospholipid hydroperoxides. Increasing evidences suggest that PRDX6 is involved in the pathogenesis of atherosclerosis and T2DM, but its role in the etiopathology of obesity and its complications is still not known. Therefore, in the present study, we sought to investigate this association by using PRDX6 knockout mice (PRDX6^−/−^). Metabolic parameters, like carbon dioxide (VCO_2_) production, oxygen consumption (VO_2_), and the respiratory exchange ratio (RER), were determined using metabolic cages. Intraperitoneal insulin and glucose tolerance tests were performed to evaluate insulin sensitivity and glucose tolerance, respectively. Liver and pancreas histochemical analyses were also evaluated. The expression of enzymes involved in lipid and glucose metabolism was analyzed by real-time PCR. Following 24 weeks of high-fat-diet (HFD), PRDX6^−/−^ mice showed weight gain and higher food and drink intake compared to controls. VO_2_ consumption and VCO_2_ production decreased in PRDX6^−/−^ mice, while the RER was lower than 0.7 indicating a prevalent lipid metabolism. PRDX6^−/−^ mice fed with HFD showed a further deterioration on insulin sensitivity and glucose-stimulated insulin secretion. Furthermore, in PRDX6^−/−^ mice, insulin did not suppress adipose tissue lipolysis with consequent hepatic lipid overload and higher serum levels of ALT, cholesterol, and triglycerides. Interestingly, in PRDX6^−/−^ mice, liver and adipose tissue were associated with proinflammatory gene upregulation. Finally, PRDX6^−/−^ mice showed a higher rate of nonalcoholic steatohepatitis (NASH) compared to control. Our results suggest that PRDX6 may have a functional and protective role in the development of obesity-related metabolic disorders such as liver diseases and T2DM and may be considered a potential therapeutic target against these illnesses.

## 1. Introduction

Obesity is a chronic disease characterized by higher levels of insulin resistance associated to an enhanced risk of developing type 2 diabetes mellitus (T2DM), nonalcoholic fatty liver disease (NAFLD) [1], cardiovascular complications, and premature death [2]. Higher fat depots, in fact, are associated with increased levels of insulin resistance, impaired glucose tolerance, T2DM, and dyslipidemia [3]. However, even if several hypotheses and theories have been postulated so far, the precise mechanisms underlying the pathophysiological link between obesity and insulin resistance and their correlated diseases have not been defined yet. Among them, enhanced liver storage of free fatty acids, increased oxidative stress levels, and activation of proinflammatory response have been demonstrated to play an important role in the interlink between obesity, insulin resistance, and their complications [4]. In overnutrition conditions such as obesity, an excess of lipids might accumulate in nonadipose tissue, which have a reduced capacity of triacylglycerol (TAG) storage. This phenomenon might increase oxidative stress as reported in the liver of obese patients with steatosis [5].

Recently, we demonstrated that peroxiredoxin 6 knockout mice (PRDX6^−/−^) developed a phenotype similar to an early stage of T2DM and were characterized by an impairment in muscle insulin sensitivity and glucose-stimulated insulin secretion [6]. Moreover, the same animals showed a hepatic proinflammatory state with typical nonalcoholic fatty liver disease (NASH) morphological features [6]. Our results innovatively suggested as PRDX6 could be involved in the complex interaction between the regulation of glucose homeostasis, lipid metabolism, and inflammatory response.

PRDX6 belongs to the peroxiredoxins (PRDXs), a family of antioxidant enzymes able to catalyze the reduction of hydrogen peroxide (H_2_O_2_), organic peroxides (ROOR′), and peroxynitrite (ONOO-) [7, 8]. Among those, PRDX6 is the only bifunctional enzyme which acts as glutathione peroxidase and phospholipase A2 (PLA2) and is able to hydrolyze phospholipids [9]. PRDX6 is widely expressed in all tissues, reaching higher levels of expression in the liver, pancreas, and kidneys [10]. Interestingly, PRDX6 concentrations are higher in hepatocytes, serving as oxidant scavengers against liver reactive oxygen species (ROS) [10].

Supporting the hypothesis that PRDX6 may be pivotal in the physiological link between glycemic and lipid components of metabolism, another study, using PRDX6 mice fed with a high-fat diet (HFD), demonstrated that these rodents were more susceptible in developing atherosclerosis compared to controls [11]. PRDX6^−/−^ mice exposed to an atherogenic diet showed higher levels of plasma lipid hydroperoxides and a consequent increase in the macrophage oxidation of low-density lipoproteins (LDL) [11], since LDL oxidation is peroxide-dependent [12]. However, despite these evidences, so far, the role of PRDX6 in the modulation of adipose tissue functionality, dyslipidemia, and progression of fatty liver diseases has not been investigated. In the present study, we analyzed the impact of PRDX6 in these processes by using a model of PRDX6^−/−^ mice in response to HFD for 24 weeks.

## 2. Materials and Methods

### 2.1. Experimental Animals

Animals were maintained in a temperature-controlled room on a 12 h : 12 h light-dark cycle with access to food and water ad libitum unless otherwise noted. Mice were fed either with standard chow diet (SCD) (Mucedola Srl, Settimo Milanese, Italy, code 4RF18) with 10% calories from fat or with HFD (58% fat, 25.5% carbohydrate, and 16.4% protein—Research Diets, Inc., NJ, USA, code D12331). Male and female PRDX6^−/−^ mice were gently provided by Professor Xiaosong Wang [10]. C57BL/6J mice were purchased from the Jackson Laboratory (Bar Harbor, Maine, USA). Genotyping of the animals was performed using DNA extracted from a small piece of tail (3-5 mm). The following primers were used: 0366 5′-CTT TGA ACA GAA CCA GGC AGG-3′, 0368 5′-CAG GAT GGA GCC TCT ATG CC-3′, and 0369 5′-TGG CTT CTG AGA CGG AAA GAA-3′. The study was approved by the University of Rome “Tor Vergata” Animal Care and Use Committee.

### 2.2. Glucose and Insulin Tolerance Test

Mice were fasted for 16 hours (h) and an Intraperitoneal Glucose Tolerance Test (IPGTT) was administered by intraperitoneal injection of 2 g/kg glucose. Blood samples were taken from the retroorbital capillary plexus at the Times (*T*) of 0, 15, 30, 60, 90, and 120 min, and glycaemia was measured using an automated OneTouch LifeScan Glucometer (Milpitas, CA) [6]. Insulin Tolerance Test (ITT) was performed after injecting 0.75 IU/kg insulin in mice fasted for 4 h. Glucose levels were measured at *T* 0, 15, 30, and 60 min [6].

### 2.3. Insulin Measurements

Blood insulin concentrations were measured in basal conditions and after IPGTT using a Mouse Insulin ELISA Kit (Mercodia, Uppsala, Sweden) according to the manufacturer's protocol. Briefly, plasma samples were loaded on a 96-well plate coated with anti-insulin antibodies and allowed to react with insulin antibodies conjugated with peroxidase for 2 h. A simple washing step removed unbound antibodies. The bound conjugates were detected by reaction with 3,3′,5,5′-tetramethylbenzidine. Then, the reaction was stopped by adding 0.5 M H_2_SO_4_ and was read spectrophotometrically at 450 nm.

### 2.4. RNA Extraction and Real-Time PCR

Total cellular RNA isolation from the liver, skeletal muscle, and white adipose tissue was obtained using the TRIzol Reagent (Invitrogen Corp., Carlsbad, CA). The High-Capacity cDNA Archive Kit (Applied Biosystems, Foster City, CA) was used to reverse-transcribed 2 *μ*g of total RNA into cDNA. Qualitative real-time polymerase chain reaction (RT-PCR) was performed using an ABI PRISM 7500 System and TaqMan reagents (Applied Biosystems). mRNA expression was monitored using commercial primers that were purchased from Life Technologies: sterol regulatory element binding transcription factor 1 (SREBP1-c)—Mm00550338_m1; TNF receptor superfamily member 6 (FAS)—Mm01204974_m; CD36—Mm01135198_m1; patatin-like phospholipase domain containing 2 (PNPLA2)—Mm00503040_m1; acyl-coenzyme A oxidase 1, palmitoyl (Acox-1)—Mm01246834_m; carnitine palmitoyl transferase 1 a (Cpt1-*α*)—Mm01231183_m1; phosphoenolpyruvate carboxykinase 1 (Pepck)—Mm01247058_m1; glucose 6 phosphatase, catalytic subunit 3 (G6P)—Mm00616234_m1; CD68—Mm03047340_m1; EGF-like module containing, mucin-like, hormone receptor-like sequence 1 (F4/80)—Mm00802529_m1; integrin alpha X (Cd11c)—Mm00498698_m; CD19—Mm00515420_m1; CD3—Mm00442746_m1; chemokine (C-C motif) ligand 3 (MCP-1)—Mm00441259_g1; chemokine (C-X-C motif) ligand 1 (KC)—Mm04207460_m; interleukin 1 beta (IL-1*β*)—Mm00434228_m; interleukin 10 (IL-10)—Mm00439614_m; tumor necrosis factor alpha (TNF-*α*)—Mm00443258_m1; interleukin 6 (IL-6)—Mm00446190_m1; interleukin 21 (IL-21)—Mm00517640_m1; adiponectin—Mm00456425_m1; leptin—mM00434759-m1; chitinase-like 3 (YM1)—Mm00657889_mH; C-type lectin domain family 10, member A (Mgl1)—Mm00546124_m1; macrophage galactose N-acetyl-galactosamine-specific lectin 2 (Mgl2)—Mm00460844_m1; and arginase, liver (Argl)—Mm00475988_m1. Gene expression was calculated using the comparative ΔΔC_T_ method, and the values were expressed as 2^−ΔΔC_T_^ (Livak KJ, Schmittgen TD). Analysis of relative gene expression data using real-time quantitative PCR and the 2^−ΔΔC_T_^ method [13]. All results were normalized using *β*-actin.

### 2.5. Histological Evaluation of Nonalcoholic Steatohepatitis and Pancreatic Islets

To obtain the percentage of islets and number of pancreatic islets, a digital camera (Dxm1200F, Nikon Italia SpA, Milan, Italy) and the Scion Image software (Scion Corporation, Frederick, MD, USA) at a 20x magnification were used. Mice liver were fixed in 10% formalin overnight, embedded in paraffin, cut at 4 *μ*m thickness, and stained with haematoxylin and eosin. Steatosis score was evaluated as previously described [14]. The number of pancreatic islets and percentage of islet area were calculated for each pancreas on at least 5 serial sections at intervals of 200 *μ*m. All analyses were performed in a blinded fashion by two different pathologists, with an interobserver variability less than 5%.

### 2.6. Metabolic Studies

Metabolic studies were performed using the LabMaster system (TSE Systems, Bad Homburg, Germany) [15]. All mice used were acclimatized in metabolic cages for 48 h before measurements of each parameters with ad libitum access to food and drink. VCO_2_ and VO_2_ were determined using O_2_ and CO_2_ sensors calibrated against a standard gas mix containing defined quantities of O_2_ and CO_2_. The ratio between VCO_2_ and VO_2_ indicates the respiratory exchange ratio (RER) [15]. Animal activity was expressed as all of the movement recorded across the *X* and *Y* axes [16]. Daily food and drink intake were monitored by weighing food hoppers and drink bottles. All the measurements were taken every 15 min for a total of 24 h.

### 2.7. Total Ketone Body Measurement

Total serum ketone body (TKB) level was obtained using a ketone body assay (Sigma-Aldrich, St. Louis, MO, USA). Briefly, 5 *μ*l of blood sample and standard solution were added to each of the wells. The levels of acetoacetic acid (AcAc) and 3-hydroxibutyric acid (BOH) were determined using the reaction catalyzed by 3-hydroxybutyrate dehydrogenase (HBDH) in which the change in absorbance, measured at 340 nm, is directly related to AcAc and BOH concentration [17]. TKB concentration was calculated using the following formula: TKB = (AcAc) + (BOH).

### 2.8. Free Fatty Acid Quantification

Total serum free fatty acid (FFA) level was obtained using the FFA Quantification Assay Kit (Abcam). According to the manufacturer's protocol, FFA were converted to their CoA derivatives and oxidized with the generation of color that was read at *λ* = 570 nm.

### 2.9. Blood Biochemistry

Serum cholesterol, triglyceride, alanine aminotransferase (ALT), aspartate aminotransferase (AST), and HDL cholesterol levels were measured by the Keylab System (BPC Biosed S.r.l., Rome Italy) [18]. Very low-density lipoproteins (VLDL) were calculated using the following formula: triglycerides/5 [19].

### 2.10. Statistical Analysis

All data are expressed as mean ± standard error of the mean (SEM). Statistical analysis was performed using unpaired one-tailed Student's *t* test or two-way analysis of variance (ANOVA) followed by Bonferroni's post hoc test. Results were analyzed using GraphPad Prism 5 (La Jolla, CA, USA), and a *p* < 0.05 was considered statistically significant.

## 3. Results

### 3.1. Basal Metabolism Decreases in Mice Lacking PRDX6 after HFD

**Figure 1:**
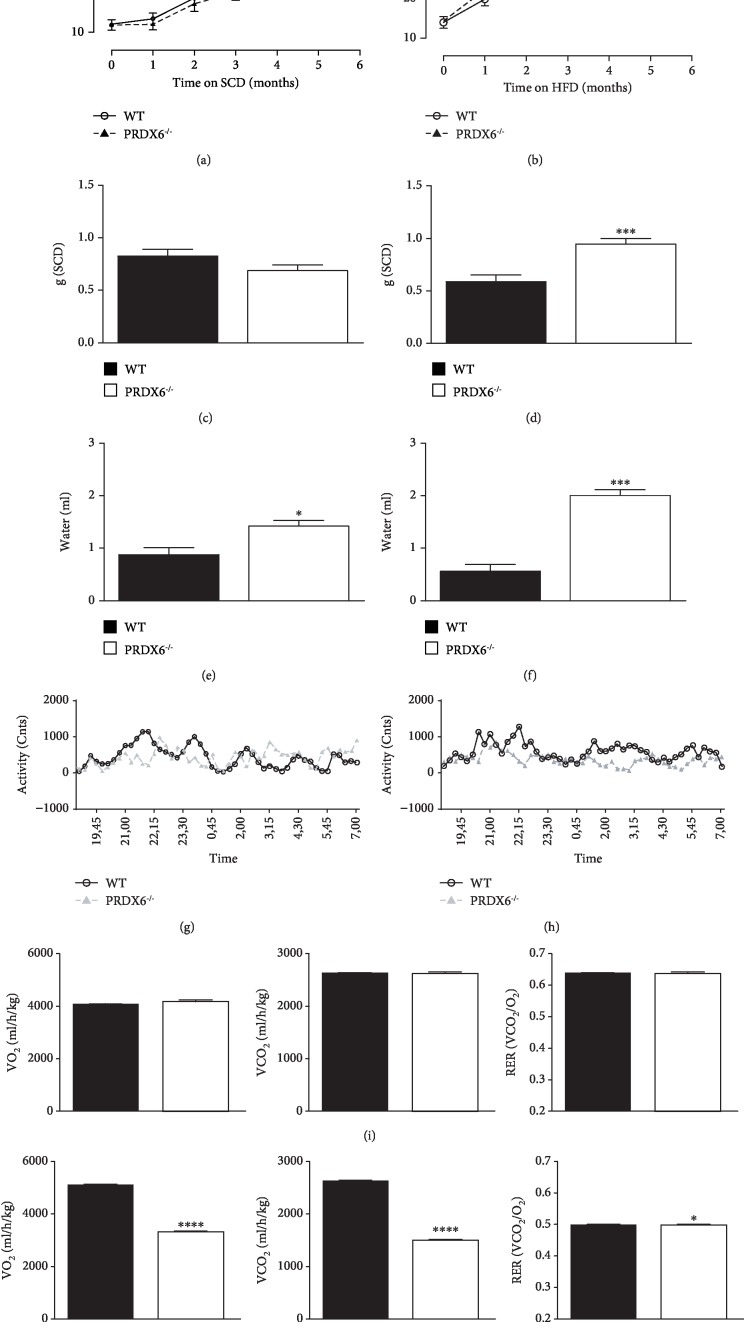
PRDX6^−/−^ mice on a HFD exhibit a different metabolic response compared to WT mice. (a and b) Body weight curves of age-matched WT (open circle, solid line) and PRDX6^−/−^ mice (filled triangle, dashed line) fed with SCD (a) and HFD (b) for 24 weeks (*n* = 15 mice per group). Measurements of food (c and d) and drink (e and f) intake obtained using metabolic cages in WT (black bar) and PRDX6^−/−^ mice (white bar) fed with SCD (c and e) and HFD (d and f) (*n* = 5 mice per group). (g and h) Horizontal and vertical movement of WT (open circle, solid line) and PRDX6^−/−^ mice (filled triangle, dashed line) fed with SCD (g) and HFD (h) for 24 weeks (*n* = 5 mice per group). (i) Measurement of gas exchange (VO_2_ and VCO_2_) and RER (VCO_2_/VO_2_) in WT (black bar) and PRDX6^−/−^ mice (white bar) fed with SCD (*n* = 5 mice per group). (j) VO_2_, VCO_2_, and RER calculation in WT (black bar) and PRDX6^−/−^ mice (white bar) after 24 weeks on HFD (*n* = 5 mice per group). The results are means ± SEM. Statistically significant differences between PRDX6^−/−^ mice and WT mice are indicated; ^∗^*p* < 0.05, ^∗∗^*p* < 0.005, ^∗∗∗^*p* < 0.0005, and ^∗∗∗∗^*p* < 0.00005.

First, since PRDX6 has an important role in regulating glucose homeostasis [6], we tested whether differences in basal metabolism were present between PRDX6^−/−^ and WT (wild type) mice. To reach our objective, metabolic cages have been utilized. PRDX6^−/−^ mice fed with SCD did not differ from WT mice in body weight and food intake during the follow-up (24 weeks) (Figures 1(a) and 1(c)). The activity, measured on the horizontal and vertical movements, was also similar for both groups fed with SCD (Figure 1(g)), suggesting that no differences in weight were associated with no differences in activity. PRDX6^−/−^ mice fed with SCD, however, showed a significant increase in drink intake compared with WT (*p* < 0.05) (Figure 1(e)).

Conversely, during the same time span of follow-up, PRDX6^−/−^ mice fed with HFD presented a significantly higher increase in weight compared to WT mice (Figure 1(b)) that was already evident after one month of diet (*p* < 0.004) (Figure 1(b)). Gain of weight of PRDX6^−/−^ mice compared to WT mice was related to more food (*p* < 0.0005) (Figure 1(d)) and drink intake (*p* < 0.0005) (Figure 1(f)). Horizontal and vertical movements in PRDX6^−/−^ mice fed with HFD were significantly reduced compared to WT (*p* < 0.0005) fed with the same diet (Figure 1(h)), suggesting an impairment in physical activity in HFD-feeding PRDX6^−/−^ mice.

After 24 hours in metabolic cages, no differences in VO_2_, VCO_2_, and RER were observed between PRDX6^−/−^ and WT mice fed with SCD (Figure 1(i)). However, after HFD, indirect calorimetric measurement reported that PRDX6^−/−^ mice had a significant decrease in VO_2_ consumption (*p* < 0.0001) and VCO_2_ (*p* < 0.0001) production and an increase in RER (*p* < 0.05) compared to WT mice. Both mice strains had a decrease in RER when fed with HFD compared to SCD, but PRDX6^−/−^ mice had relatively less fat oxidation compared to WT mice, since RER values were significantly higher (*p* < 0.05) (Figure 1(j)), confirming the hypothesis of a lower basal metabolism in these animals after an intake of a high amount of fat. However, RER values lower than 0.7 suggested the synthesis of carbohydrates or ketone body metabolism in mice lacking of PRDX6 [20].

### 3.2. Function and Anatomical Structure of Pancreatic Beta Cells Are Impaired in PRDX6^−/−^ Mice Fed with HFD

**Figure 2:**
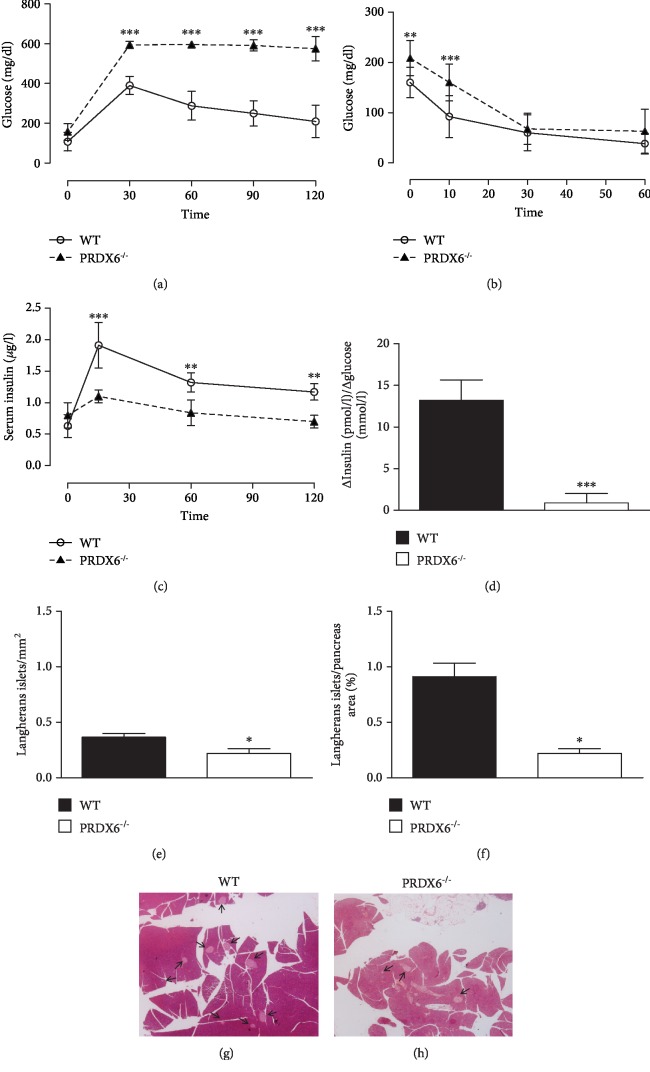
PRDX6^−/−^ mice display impaired glucose tolerance and insulin sensitivity following 24 weeks on HFD. (a) After an overnight fast, an IPGTT was performed by injecting intraperitoneally 2 g/kg body weight of D-glucose. Blood samples were taken from the retroorbital cavity of the mice at 0, 15, 30, 60, 90, and 120 minutes after the injection of D-glucose. WT mice (open circle, solid line) and PRDX6^−/−^ mice (filled triangle, dashed line) (*n* = 12 mice per group). (b) ITT was performed by intraperitoneal injection of insulin at 0.75 IU/kg body weight following 4 hours of fasting. After the injection of insulin, blood glycaemia was measured at 0, 15, 30, and 60 minutes. WT mice (open circle, solid line) and PRDX6^−/−^ mice (filled triangle, dashed line) (*n* = 12 mice per group). (c) The quantitative assessment of plasma insulin secretion during the IPGTT were analyzed by ELISA at 0, 15, 60, and 120 minutes after glucose injection. WT mice (open circle, solid line) and PRDX6^−/−^ mice (filled triangle, dashed line) (*n* = 5 mice per group). (d) Calculation of insulinogenic index as delta insulin to the delta glucose ratio (*∆I*0‐15/*∆G*0‐15) in WT (black bar) and PRDX6^−/−^ mice (white bar) (*n* = 5 mice per group). (e and f) Valuation of islet density (e) and size (f) in WT mice (black bars) and PRDX6^−/−^ mice (white bar). (g and h) Representative microphotographs of pancreatic islet of Langerhans after 24 weeks on HFD in WT mice (g) and PRDX6^−/−^ mice (h). The images are shown at 20x magnification. The results are expressed as means ± SEM. ^∗^*p* < 0.05, ^∗∗^*p* < 0.005, and ^∗∗∗^*p* < 0.0005.

Mice lacking PRDX6 but fed with SCD develop a mild form of DM, mainly linked to higher levels of insulin resistance associated with a defect of glucose-stimulated insulin secretion (GSIS) [6]. In order to further understand the impact of this antioxidant enzyme on the regulation of glycemic homeostasis and maintenance of a functional pancreatic *β*-cell mass, we further investigated the metabolic response of PRDX6^−/−^ and WT mice after 24 weeks of treatment with HFD. Both PRDX6^−/−^ and WT mice underwent IPGTT (Figure 2(a)) and ITT (Figure 2(b)). After IPGTT, PRDX6^−/−^ mice had significantly higher levels of blood glucose than WT mice in all time points evaluated (*p* < 0.05 at 0 min, and *p* < 0.001 at 30, 60, 90, and 120 min) (Figure 2(a)). Furthermore, PRDX6^−/−^ mice after an ITT test showed a reduced insulin response compared to WT mice (*p* < 0.01 and *p* < 0.001 at 0 and 15 min, respectively) (Figure 2(b)), indicating that PRDX6^−/−^ mice with higher fasting glucose levels are insulin resistant. Measurement of insulin secretion during IPGTT were significantly reduced at 15 (*p* < 0.001), 60, and 120 min (*p* < 0.01) in PRDX6^−/−^ mice compared to WT mice (Figure 2(c)). The insulinogenic index was also calculated to investigate the function of pancreatic *β*-cells at 15 min, resulting in lower levels in PRDX6^−/−^ mice (*p* < 0.005) (Figure 2(d)), further confirming the impairment of *β*-cell function in the absence of PRDX6, as previously reported [6].

Similarly, histochemical analysis of pancreas established previous findings [6], showing that PRDX6^−/−^ mice have a significantly lower number (*p* < 0.05) and size (*p* < 0.05) of pancreatic islets compared to WT mice (Figures 2(e), 2(f), 2(g), and 2(h)). These results suggest that the absence of this specific antioxidant enzyme may influence both the function and the anatomical structure of pancreatic islets in mice fed with a normal diet but with a further increase in HFD.

### 3.3. Lack of PRDX6 Impaired Lipid and Glucose Metabolism

**Figure 3:**
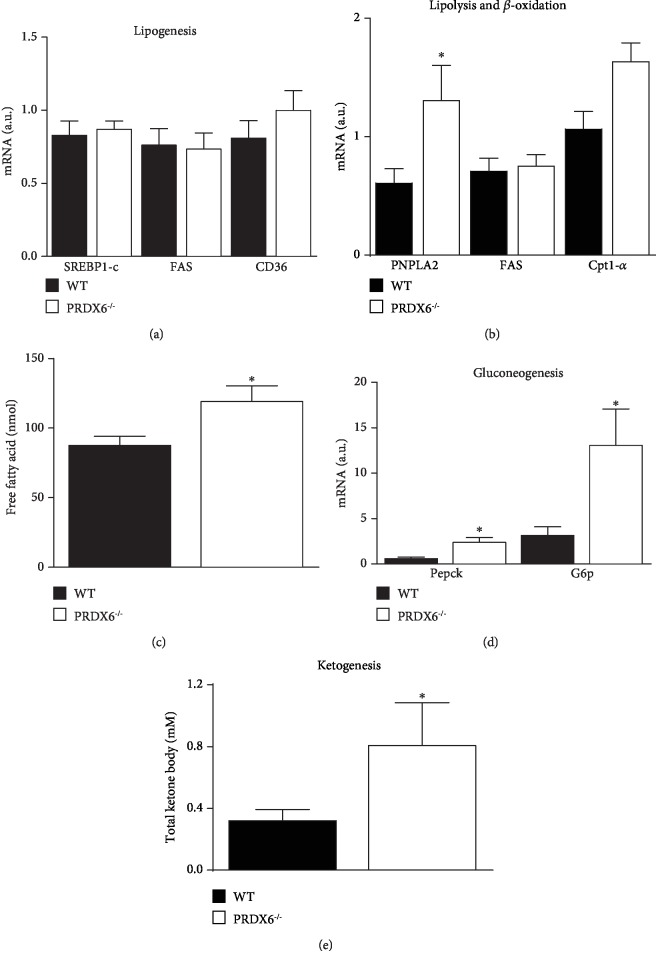
PRDX6^−/−^ mice show an upregulation in the release of free fatty acid from adipose tissue, liver gluconeogenesis, and ketogenesis. mRNA expression of genes involved in (a) lipid biogenesis (SREBP1-c, FAS, and CD36), (b) lipolysis and *β*-oxidation (PNPLA2, Acox-1, and Cpt1-*α*), (c) total serum FFA concentration, (d) liver gluconeogenesis (Pepck, G6P), and (e) total serum ketone body concentration that was calculated using the following formula: TKB = (AcAc) + (BOH) (*n* = 8 mice per group). a.u.: arbitrary units. The results are expressed as means ± SEM. Statistically significant differences between PRDX6^−/−^ mice and WT mice are indicated; ^∗^*p* < 0.05.

Lipid metabolism impairment and FFA trafficking from adipose tissue to liver are key events in the development of T2DM and NAFLD [21]. Therefore, we analyzed the mRNA expression of genes involved in adipose tissue lipid biogenesis and transport (SREBP1-c, FAS, and CD36) (Figure 3(a)), lipolysis (PNPLA2) (Figure 3(b)), and *β*-oxidation (Acox-1 and Cpt1-*α*) (Figure 3(b)). PRDX6^−/−^ mice displayed significantly increased levels of PNPLA2 compared to WT mice (*p* < 0.05) (Figure 3(b)). PNPLA2 is the principal enzyme catalyzing the initial step of triglyceride hydrolysis in adipocytes [22, 23]. Indeed, the serum levels of FFA were significantly higher in PRDX6^−/−^ than WT mice (*p* < 0.05) (Figure 3(c)). Thus, the higher expression of PNPLA2 in PRDX6^−/−^ mice may enhance the hydrolysis of triglycerides, increasing FFA blood levels, the risk of its accumulation in the liver, and the development of NAFLD. In obese patients with T2DM, higher levels of circulating FFA and/or increased rate of lipolysis lead to gluconeogenesis [24]. Moreover, a massive mobilization of FFA from adipose tissue results in an increased production of ketone bodies derived from FFA oxidation into AcAc and BOH in the liver, and in smaller proportion, in muscle [25]. Therefore, a link between FFA, liver metabolism gluconeogenesis, and ketogenesis is well established.

In our model, a lack of PRDX6 in an animal fed with HFD was associated with a significant upregulation of gluconeogenesis in comparison with WT mice (Figure 3(d)). Indeed, mRNA expression of Pepck (*p* < 0.05) and G6P (*p* < 0.05), the two key enzymes which regulate the first and the latest reaction of gluconeogenesis [26], was higher in PRDX6^−/−^ than in WT mice.

To determine the total serum ketone bodies, the amount of AcAc and BOH was calculated. PRDX6^−/−^ mice fed with HFD showed an increased production of blood total ketone bodies compared to WT mice (*p* < 0.05) (Figure 3(e)), suggesting that high levels of FFA released from adipose tissue may have a significant effect on ketogenesis in these animals. This is in agreement with the RER levels < 0.7, which suggested an increased ketone body metabolism.

### 3.4. Increased Risk of Developing NASH in PRDX6 Knockout Mice after a Proinflammatory Diet

**Figure 4:**
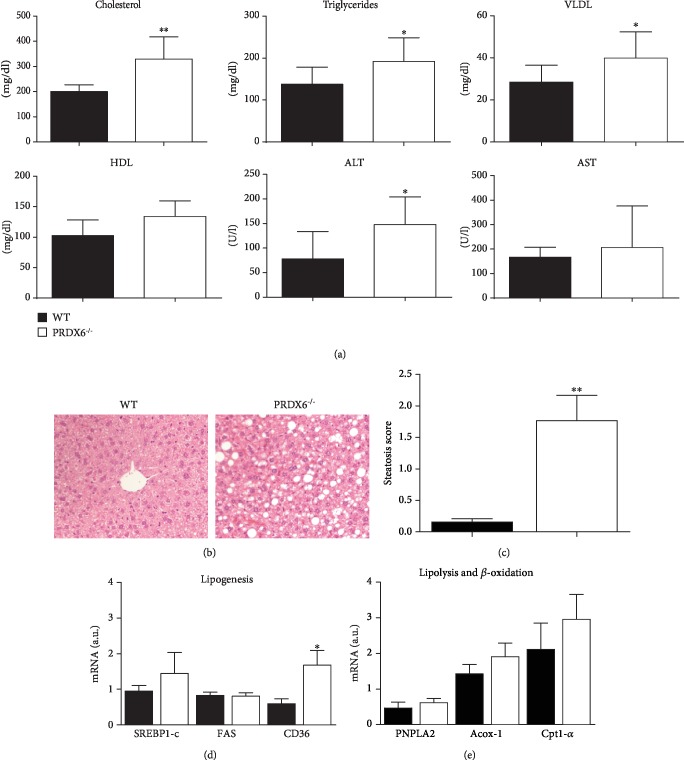
PRDX6 deficiency induces hepatic steatosis on HFD. (a) Measurement of cholesterol, triglycerides, VLDL, HDL, ALT, and AST. Following 24 weeks on HFD, fasting serum samples in WT (black bar) and PRDX6^−/−^ mice (white bar) were taken (*n* = 5 mice per group). (b) Microscopic analysis of the liver section in WT and PRDX6^−/−^ mice upon HFD for 24 weeks. The images are shown at 20x magnification. (c) Steatosis score valuation in WT (black bar) and PRDX6^−/−^ mice (white bar) (*n* = 5 mice per group). mRNA expression of genes involved in (d) lipid biogenesis (SREBP1-c, FAS, and CD36) and (e) lipolysis and *β*-oxidation (PNPLA2, Acox-1, and Cpt1-*α*). The results are expressed as means ± SEM. Statistically significant differences between PRDX6^−/−^ mice and WT mice are indicated; ^∗^*p* < 0.05 and ^∗∗^*p* < 0.005.

NAFLD is the most common liver disease in obese and T2DM patients, and its main feature is the accumulation of triglycerides in hepatocytes with consequent steatosis [27]. Often, this condition evolves to NASH [28]. Based on the data reported above, we aimed to determine the role of PRDX6 on NAFLD and its progression to NASH. We evaluated serum levels of ALT, AST, cholesterol, VLDL, and triglycerides, and the liver histological analysis in PRDX6^−/−^ and WT mice fed with HFD. Circulating serum levels of cholesterol and VLDL in PRDX6^−/−^ mice were raised compared to WT mice (*p* < 0.005 and *p* < 0.05, respectively) (Figure 4(a)). Instead, HDL cholesterol blood concentrations were similar between the two groups of animals (Figure 4(a)). Furthermore, the levels of triglycerides increased in PRDX6^−/−^ in comparison to WT mice (*p* < 0.05). As an important marker of hepatic injury, ALT level was higher (*p* < 0.05) in PRDX6^−/−^ mice compared to WT mice (Figure 4(a)). No significant change was evident in the AST level between the two groups of animals (Figure 4(a)).

Histological analysis of the liver in mice fed a HFD and obese mice showed a histological pattern characterized by microvesicular steatosis in WT mice, as expected, while a microvesicular and macrovesicular state of steatosis prevailed in PRDX6^−/−^ mice, with signs of lobular inflammation and hepatocellular ballooning (Figure 4(b)). Indeed, the steatosis score in PRDX6^−/−^ mice was significantly higher compared to WT mice (Figure 4(c)) (*p* < 0.005).

To further investigate whether the higher level of steatosis present on PRDX6^−/−^ mice depended on the increased lipid metabolism or by the increased hepatic uptake of FFA, genetic expression (mRNA) of the main enzymes involved in these pathways were evaluated (SREBP1-c, FAS, and CD36). Interestingly, the expression of CD36, which mediates the hepatic uptake of FFA [29], increased in PRDX6^−/−^ mice (*p* < 0.05) (Figure 4(d)). In addition, the mRNA levels of enzymes involved in lipolysis and *β*-oxidation (PNPLA2, Acox-1, and Cpt1-*α*) were examined. Genetic expression of PNPLA2 in the liver of PRDX6^−/−^ mice did not differ compared to WT mice (Figure 4(e)), suggesting a tissue-specific pattern of expression of this gene. Similar data were present for Cpt1-*α*, the protein responsible for the transport of fatty acyl-CoA through the inner mitochondrial membrane [30], and Acox-1, the enzyme that catalyzes the first step in peroxisomal *β*-oxidation [31] (Figure 4(e)). Based on these results, we may suggest that the steatosis state in the absence of PRDX6 and on HFD could be a consequence of hepatic lipid overload.

### 3.5. Effect of PRDX6 on the Inflammatory State

HFD is a proinflammatory input which mimics the obesity inflammatory pathological background [32]. By measuring the mRNA expression of cytokines and chemokines, as principal markers of inflammation in the pivotal tissues (adipose tissue, liver, skeletal muscle), we sought to understand the role of PRDX6 in this process. The list of markers measured by real-time PCR is reported in Table 1. After 24 weeks on HFD, PRDX6^−/−^ mice showed a significant upregulation (*p* < 0.05) in the expression of genes coding for TNF-*α*, IL-1*β*, IL-6, and MCP-1 compared to WT mice, measured in the adipose tissue and in the liver (Table 1). In adipose tissue, a lack of PRDX6 also had a significant impact in leptin synthesis (*p* < 0.005), whereas the adiponectin level did not change (Table 1).

In the skeletal muscle, the PRDX6^−/−^ mice only showed a higher expression for TNF-*α* and MCP-1 compared to WT, whereas the other cytokines did not change (Table 1), suggesting a specific effect of these enzymes in the regulation of the inflammatory process, which deserves further investigation.

## 4. Discussion

In the present study, innovatively, we reported a pivotal role of PRDX6 in the pathogenesis of obesity and related liver diseases, particularly nonalcoholic fatty liver disease and nonalcoholic steatohepatitis. By using a model of PRDX6 knockout mice fed a HFD for 24 weeks, we demonstrated that a lack of this enzyme was associated with a significant weight gain due to increased food intake, and decreased physical activity. Drink intake was also enhanced in these mice, probably due to markedly elevated glucose levels, glycosuria, and osmotic diuresis. An increase in adipose tissue leptin production was observed, possibly linked to higher levels of hypothalamic leptin resistance [33], or to higher signals of energy repletion, since for unknown reasons, PRDX6^−/−^ mice could be more exposed to an increase of palatable food in response to HFD. A proteomic study conducted in adipose tissue of leptin-deficient (ob/ob) mice treated with leptin recognized PRDX6 as one among 12 functional proteins that by regulating mitochondrial physiology and oxidative stress were implicated in obesity mechanisms [34].

HFD-fed PRDX6^−/−^ mice had reduced basal metabolism compared to SCD-fed mice; on the contrary, RER was significantly higher suggesting an increased synthesis of carbohydrates and ketone body metabolism [20], confirmed by higher glucose and ketone blood concentrations. Moreover, it might be also linked to higher weight gain in this strain due to a lesser capacity to oxidize fat compared to WT mice [35]. Furthermore, glucose-induced insulin release was reduced in PRDX6^−/−^ mice along with a lower insulinogenic index. Lower insulin levels may be correlated with the observed increase in food intake in these mice, since insulin action in hypothalamic nuclei, like the arcuate nucleus [36] (central regulatory of feeding and energy homeostasis) when decreased, affects appetite and consequently body weight [37]. Finally, in the absence of PRDX6, pancreatic islets were reduced in number and size compared to WT highlighting a relevant role of PRDX6 in maintaining the structure of pancreatic *β*-cells [38]. Since PRDX6 is among the most important antioxidant enzymes in pancreatic *β*-cells, its absence can influence the cellular redox system and the insulin secretion as well. This has been hypothesized as the main mechanism underlying pancreatic dysfunction linked with a lack of PRDX6 since this enzyme may play a role at different cellular levels in controlling ROS production and therefore cellular functions, such as insulin secretion. In fact, a direct action of PRDX6 on mitochondria clearance [39] and in the modulation of electron transport chain [40] has been proposed, processes that are directly implicated with oxidative stress and oxidative stress-related diseases.

Several evidences outline a role of PRDXs in the modulation of hepatic functions: PRDX1 expression was decreased in the liver of HFD-fed mice [41], PRDX4 transgenic mice were resistant to hepatic steatosis, and insulin resistance increased in response to HFD [42]. We found that PRDX6^−/−^ mice have higher levels of insulin resistance [6], and PRDX6 protein and mRNA expression decreased in the liver of a mouse model of ethanol consumption [43]. In this study, we reported that HFD-fed PRDX6^−/−^ mice showed advanced liver dysfunction with signs of lobular inflammation and hepatocellular ballooning indicating a condition of NASH, not present in WT mice. Liver metabolism was also affected by a significant upregulation in gluconeogenic and CD36 gene expression, which contribute to hepatic lipid accumulation in the presence of increased FFA supply, as documented by higher VLDL and FFA blood levels observed in PRDX6^−/−^ mice. CD36, a scavenger receptor enhanced in the liver of HFD-induced obese mice [44], plays an important role in liver dysfunction progression [45]. Hepatic resident macrophages expressing CD36 contribute to the accumulation of a liver fat depot [46]. Additionally, CD36 recognizes specific fatty acids such as palmitate and oleic acids, activating intracellular signaling pathways which lead to proinflammatory cytokine production [47]. According to these findings, we reported that a lack of PRDX6 was associated with a higher level of inflammatory parameters in all tissues investigated, and in particular, the liver showed high levels of dendritic, T cell, and macrophage marker expression and increased levels of cytokines, such as IL-1*β*, TNF-*α*, and IL-6. Among those, TNF-*α*, specifically, has been shown to be directly involved in the development of hepatic steatosis and insulin resistance [48]. These results suggested that mice lacking PRDX6 were more prone to develop a severe form of liver dysfunction, such as NASH, highlighting the role of this antioxidant enzyme in the pathogenesis of metabolic liver disease in obese and diabetic conditions. The anti-inflammatory properties of PRDX6 were already reported in corneal cells, where the administration of this enzyme was able to reduce inflammation and apoptosis [49]. A recent study revealed that TNF-*α* or INF-*γ* decreased both the mRNA and protein expression of PRDX6 in pancreatic *β* cells, further confirming the crosslink between this antioxidant enzyme and tissue-specific inflammatory processes [50].

Obesity, a condition with chronic low-grade inflammation, is strictly linked with liver abnormalities such as NAFLD. According to the traditional “two-hit hypothesis,” NAFLD can evolve to NASH, when after triglyceride accumulation (1 hit) follows the induction of an inflammatory pathway and the overproduction of ROS which leads to steatohepatitis (2 hits) [51]. However, evidences now indicate that in conditions like obesity and insulin resistance, the increased flux of FFA from adipose tissue is the main cause of liver injury, through oxidative stress and inflammation, that progress to NASH [52]. In insulin resistance conditions, the antilipolytic effect of insulin on adipose tissue is decreased [53]. In association, the high PNPLA2 gene expression levels found in adipose tissue of PRDX6^−/−^ mice may partially explain the increased levels of TG and liver lipid accumulation. Moreover, FFA released from adipose tissue cross the hepatic plasma membrane directly for diffusion or through the CD36 protein, the levels of which have been found enhanced in the liver of PRDX6^−/−^ mice and in patients with NAFLD, and its expression is correlated with the hepatic lipid content [54]. Therefore, the increase in hepatic lipid content observed in these animals is mainly due to an increase in the incoming of lipids in the liver. Cholesterol and VLDL blood concentrations were increased in PRDX6^−/−^ mice, together with higher levels of ALT. This is in agreement with the presence of NASH and the increase in liver lipid content of PRDX6^−/−^ mice that lead to the alteration of liver metabolism and enhancement of VLDL production and secretion. A previous study [40] has already demonstrated a protective role for PRDX6 during hepatocellular injury, likely due to its strong expression in the liver, and downregulation of PRDX6 was reported in the liver of donors after brain death associated with oxidative stress induced by ischemia and hypoxia [55]. Recently, in agreement with our finding, it has been demonstrated in the PRDX6 transgenic mouse model that PRDX6 protects against NAFLD progression, preserving mitochondrial integrity in response to HFD [56]. Taken together, all these findings suggest that increasing the activity of this antioxidant enzyme may be a novel strategy to alleviate liver damage in dysmetabolic conditions, such as obesity and T2DM.

The strengths of the present study are as follows: the use of a novel genetic model of PRDX6^−/−^ mice, a significantly long time of follow-up for an *in vivo* study (24 weeks), the use of proinflammatory HFD, and detailed measurement of histological and biochemical parameters. The main limitation to acknowledge for this study is that as in all genetic models of knockout animals, other unexpected compensatory or redundant antioxidant and anti-inflammatory mechanisms may be present and it is very difficult to evaluate their effects [57].

## 5. Conclusion

In conclusion, in the present study, we reported a direct role of PRDX6 in the pathological link between obesity and liver diseases by the modulation of noxious oxidative stress and inflammation processes. A better understanding of the mechanisms underlying this link may allow us to develop novel therapeutic strategies against metabolic illnesses and their complications, which may have a direct effect in terms of extending longevity and preventing obesity comorbidity. Further studies to clarify the role of PRDX6 and metabolic diseases, such as obesity and liver dysfunction, are imperative.

## Figures and Tables

**Table 1:** PRDX6^−/−^ mice develop a proinflammatory state in liver and white adipose tissue. Gene expression analysis obtained by real-time PCR in WT mice and PRDX6^−/−^ mice for leukocyte cell surface markers (CD68, F4/80, CD11c, CD19, CD3, Arg1, Mgl1, Mgl2, and YM1), chemokines (MCP-1 and Kc), and cytokines (IL-1*β*, IL-10, TNF-*α*, IL-6, and IL-21) (*n* = 8 mice per group). All the results were normalized using *β*-actin and expressed as means ± SEM. a.u.: arbitrary units; ND: not detectable; NS: not significant *p* value. ^∗^*p* < 0.05 and ^∗∗^*p* < 0.005.

	Adipose tissue			Liver			Skeletal muscle		
Gene (a.u.)	WT mice	KO mice	*p* value	WT mice	KO mice	*p* value	WT mice	KO mice	*p* value
IL-*β*	0.89 ± 0.57	1.84 ± 1.07	<0.05	2.24 ± 1.74	8.68 ± 5.47	<0.05	1.9 ± 2.29	1.91 ± 1.13	NS
IL-10	1.76 ± 1.9	1.55 ± 1	NS	0.7 ± 0.45	4.10 ± 4.42	<0.05	0.57 ± 0.28	1.18 ± 0.95	NS
TNF-*α*	1.03 ± 0.44	2.10 ± 1.35	<0.05	0.59 ± 0.39	1.82 ± 1.34	<0.05	0.88 ± 0.25	2.16 ± 0.06	<0.05
IL-6	0.86 ± 0.43	1.4 ± 0.64	<0.05	1.36 ± 0.78	4.31 ± 2.69	<0.05	1.52 ± 1.93	1.29 ± 1.17	NS
IL-21	0.88 ± 0.54	0.96 ± 0.58	NS	2.77 ± 1.7	3.39 ± 1.34	NS	ND	ND	ND
Leptin	1.2 ± 0.58	4.7 ± 2.19	<0.05	ND	ND	ND	ND	ND	ND
Adiponectin	0.46 ± 0.30	0.61 ± 0.2	NS	ND	ND	MD	ND	ND	ND
MCP-1	1.35 ± 1.57	4.88 ± 2.18	<0.05	0.62 ± 0.08	1.39 ± 0.37	<0.05	1.51 ± 0.34	3.77 ± 1.64	<0.05
Kc	1.5 ± 1.78	3.54 ± 3.99	NS	0.39 ± 0.17	7.4 ± 2.03	<0.05	0.61 ± 0.33	0.84 ± 0.36	ND
CD68	0.6 ± 0.42	1.29 ± 0.6	<0.05	1.32 ± 0.27	1.66 ± 0.2	NS	0.94 ± 0.13	1.46 ± 1.71	NS
F4/80	0.74 ± 0.32	1.45 ± 0.82	<0.05	0.84 ± 0.21	2.37 ± 0.34	<0.05	0.67 ± 0.21	0.62 ± 0.06	NS
CD11c	0.94 ± 0.55	5.87 ± 2.76	<0.05	0.37 ± 0.16	1.41 ± 0.31	<0.05	1.35 ± 0.70	2.50 ± 3.10	NS
CD19	0.51 ± 0.33	0.94 ± 0.19	<0.05	0.38 ± 0.37	0.32 ± 0.19	NS	1.88 ± 2.61	0.73 ± 0.42	NS
CD3	0.4 ± 0.35	0.6 ± 0.58	NS	0.89 ± 0.6	2.26 ± 1.01	<0.05	0.94 ± 0.41	1.01 ± 0.46	NS
Arg1	0.96 ± 0.49	1.65 ± 0.81	NS	0.77 ± 0.38	0.94 ± 0.47	NS	0.65 ± 0.42	0.73 ± 0.37	NS
Mgl1	1.57 ± 0.77	2.03 ± 0.99	NS	0.85 ± 0.39	1.72 ± 106	NS	1.37 ± 0.33	2.3 ± 0.99	NS
Mgl2	0.87 ± 0.29	0.76 ± 0.19	NS	0.99 ± 0.25	2.15 ± 2.05	NS	1.32 ± 0.41	1.78 ± 0.41	NS
YM1	1.65 ± 1.26	2.36 ± 0.5	NS	0.6 ± 0.41	2.53 ± 2.44	NS	0.27 ± 0.48	0.14 ± 0.13	NS

## Data Availability

All the data in the figures and tables used to support the findings of this study are included within the article.

## Supplementary Materials

Supplementary Materials Experimental flow diagram. In this study, WT and PRDX6^−/−^ mice were fed with HFD. After 24 weeks of HFD diet, we performed (i) measurement of glucose homeostasis by IPGTT and ITT; (ii) histochemical analysis of pancreas; (iii) evaluation of NAFLD by calculation of the steatosis score and FFA metabolism; and (iv) real-time PCR analysis of the main genes involved in lipid and glucose metabolism as well as those implicated in inflammation in the liver, skeletal muscle, and adipose tissue.
