# Supplementary material for: Peroxiredoxin 6 Is a Key Antioxidant Enzyme in Modulating the Link between Glycemic and Lipogenic Metabolism
Source: Oxid Med Cell Longev. 2019 Dec 19;2019:9685607. doi: 10.1155/2019/9685607 (PMC6948322; doi:10.1155/2019/9685607)
Supplement: Supplementary Materials — Experimental flow diagram. In this study, WT and PRDX6−/− mice were fed with HFD. After 24 weeks of HFD diet, we performed (i) measurement of glucose homeostasis by IPGTT and ITT; (ii) histochemical analysis of pancreas; (iii) evaluation of NAFLD by calculation of the steatosis score and FFA metabolism; and (iv) real-time PCR analysis of the main genes involved in lipid and glucose metabolism as well as those implicated in inflammation in the liver, skeletal muscle, and adipose tissue. [file 9685607.f1.pdf]

Experimental flow diagram

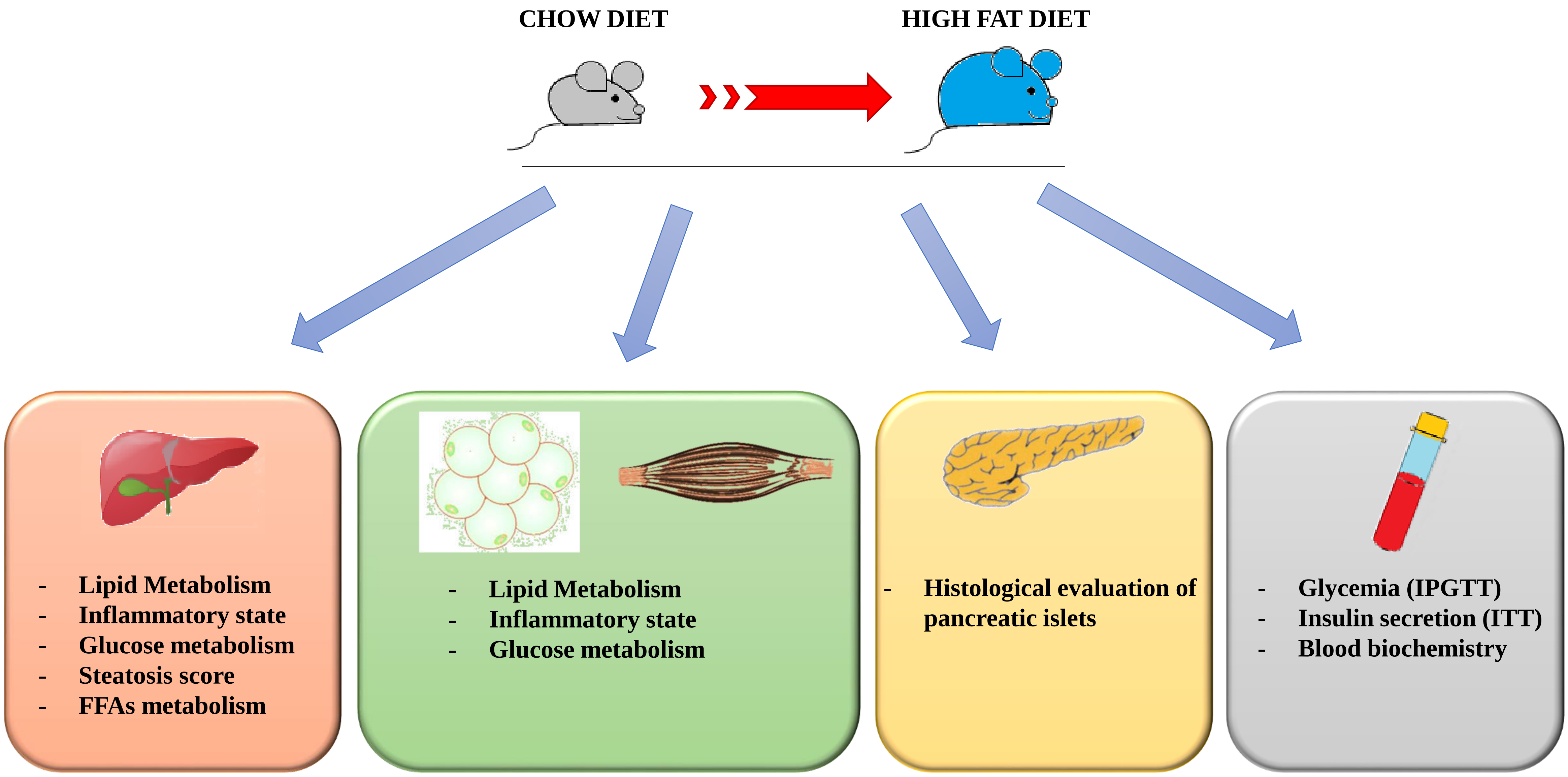
CHOW DIET
HIGH FAT DIET
- Lipid Metabolism
- Inflammatory state
- Glucose metabolism
- Steatosis score
- FFAs metabolism
- Lipid Metabolism
- Inflammatory state
- Glucose metabolism
- Histological evaluation of pancreatic islets
- Glycemia (IPGTT)
- Insulin secretion (ITT)
- Blood biochemistry
